# City-scale residential energy consumption prediction with a multimodal approach

**DOI:** 10.1038/s41598-025-88603-2

**Published:** 2025-02-13

**Authors:** Yulan Sheng, Hadi Arbabi, Wil O. C. Ward, Mauricio A. Álvarez, Martin Mayfield

**Affiliations:** 1 Present Address: School of Earth and Environment, University of Leeds, Leeds, UK; 2 School of Mechanical, Aerospace and Civil Engineering, The University of Sheffield, Sheffield, UK; 3 School of Computing and Mathematical Sciences, University of Leicester, Leicester, UK; 4 Department of Computer Science, University of Manchester, Manchester, UK

**Keywords:** Residential Energy Consumption Prediction, Energy performance certificates (EPC), Partial Dependence, AutoML, Sustainability, Energy and society, Energy efficiency

## Abstract

The key role of buildings in tackling climate change has gained global recognition. To avoid unnecessary costs and time wasted, it is important to understand the conditions and energy usage for existing housing stock to identify the most important features affecting energy consumption and to guide the relevant retrofit measures. This paper investigated how the spatial, morphological and thermal characteristics of residential houses contribute to housing energy consumption. Additionally, it presents a rapid assessment tool using minimum data input to answer two main questions: 1) *What type of properties may need retrofit?* 2) *What building elements/features may be prioritised to be retrofitted?* A case study was performed with around 143,000 residential properties in Sheffield. An automated machine approach was applied which successfully estimated the energy consumption of target buildings with an $R^2$ score of 0.828. Permutation feature importance and partial dependence of the features were examined against energy consumption. The results indicate that housing sizes and conditions of the external walls are found to be the most important features when estimating the energy consumption of residential buildings in Sheffield. Relatively larger and older detached houses in neighbourhoods with higher build density may benefit the most from home upgrading projects for energy consumption reduction.

## Introduction

### Background

Residential buildings have become one of the largest consumers of energy around the world^1^. The recent years have witnessed the growing pressure residents feel in paying energy bills, caused in part by the worldwide COVID-19 pandemic and the rapid increase in energy prices^2^. In the UK, the residential sector is the only sector that has risen in energy consumption since 2019, while other sectors: transport, industry and services, all decreased^3^. This increasing trend hints at the difficulties the UK government is currently facing in achieving its net-zero emissions goals by 2050 to tackle the climate crises.

Incentives have been introduced to mitigate the energy and environmental crisis. The UK government has proposed to raise the minimum energy standards for domestic buildings by 2030, especially privately rented houses^4^. According to the English Housing Survey (EHS) for 2022 to 2023, around 52% of existing housing stocks will require either retrofitting to meet the new standard or demolishing and reconstructing^5^.

Comparative studies conducted for retrofit and demolition have concluded retrofitting is more environmentally friendly, although it can be relatively expensive. The most common retrofitting measure used, upgrading the insulation of the external wall, can cost up to $\pounds$20,000 per home^6^. If all the properties in the UK are due to be improved to the minimum required standards, the average costs are estimated to be between $\pounds$91 and $\pounds$94 billion^5^. UK government is investing nearly $\pounds$4 billion from 2022 to 2026 to support home upgrading and retrofitting^2^. It is thereby important to understand the buildings’ current energy performance to help determine optimal retrofit measures to achieve net-zero targets.

This paper developed machine learning models for age and energy consumption prediction and further analysed the correlations between each building feature and energy usage to provide guidance on effective retrofit measure selection. This paper investigated how the spatial, morphological and thermal characteristics of residential houses contribute to housing energy consumption. We also provide a rapid assessment tool using minimum data input to answer two main questions: 1) *What type of properties may need retrofit?* 2) *What building elements/features may be prioritised for retrofit to improve energy efficiency?*

### Related work

When estimating residential energy performance, there are three approaches commonly found in the existing literature, either a data-driven approach, a physics-based approach or a hybrid method that combines the previous two approaches. Both the physics-based and hybrid approaches rely on detailed information on buildings’ thermal characteristics, such as the thermal transmittance of the building material^7^. They are usually applied in relatively small-scale studies focusing on a single building. When access to meter readings and buildings’ internal space is limited, a data-driven approach is usually applied to develop statistical or machine learning models, based on historical energy consumption data and building morphology. It has been found that, in general^8,9^:

1. Buildings constructed in similar periods tend to have similar building characteristics; and
2. Buildings with similar characteristics tend to have similar energy consumption.

Each rule suggests one main feature affecting the buildings’ energy performance. The first rule indicates the year of construction is important in energy estimation. One of the potential reasons is that, housing legislation changes regularly to comply with the housing needs and environmental concerns at that time and also what might be needed in the future. For instance, the Town and Country Planning Act issued in 1947^10^ prioritised developing single apartment blocks. The construction sector then develops homes accordingly, hence the second rule^10^.

Despite the importance of building age in inferring building energy consumption, no easily accessible complete database is often available^8^. Existing studies have attempted to infer building age from its physical features^9,11^.^8^ proposed a methodology to predict the year of construction using map data and historical satellite images. Their machine learning model used random forests and achieved 77% prediction accuracy^8^. However, their model was trained based on a relatively small number of properties (1,096) in Nottingham to predict 5 aggregated age bands covering a rather wide time span. The test samples they used were also derived from a single neighbourhood, which tends to have similar building features and construction age.

The second rule, the relationship between building characteristics and energy consumption, provides insight into how housing features can be used to estimate energy using the data-driven approach. Existing literature has experimented with a wide range of different data inputs providing such information, including data either in 2D or 3D, e.g. LiDAR point cloud^12^, text-based^13^ or image-based^14,15^. One widely used is the Energy Performance Certificate (EPC). EPC is an official document of buildings’ energy performance required for every property in the UK, similar to the Energy Star score in the USA and Diagnostic de Performance Energétique in France^16^. It follows the Standard Assessment Procedure (SAP) which calculates a rating representative of the overall building energy performance. The SAP can be considered a simplified physics-based approach in the form of a worksheet. It calculates a score based on the building specifications, such as the floor area, the standard U-values of the material used, and the average regional temperature, to a scale of 0 to 100^17^. The scores are then converted to the EPC rating, ranked from G, the least efficient, to A, the most efficient^17^.^15^ developed a workflow that uses existing EPC data to predict buildings’ energy ratings when such information is not available. Their best-performing machine learning model has achieved 88% accuracy in predicting building EPC ratings for properties in Ireland. However, there are issues with EPCs that the above studies did not take into consideration. For instance,^18^ have summarised that there are around 1.6 million properties found to be associated with multiple valid EPCs in the system. The study carried out by^19^ also revealed that the records in EPCs can be subjective to the inspectors. In their evaluation of 29 houses assessed by multiple inspectors, nearly two-thirds of the properties received ratings that differed across two EPC bands, underscoring the critical limitations in the EPC records. However, the EPC is one of the most comprehensive publicly available databases for studies related to residential properties.

Machine-learning based data-driven approach is one of the popular methods adopted by existing studies to estimate buildings’ energy performance^8,9,13,20^. However, these models were usually designed using data and algorithms chosen based on researchers’ knowledge or the ones previous studies have used, which may not be suitable when local contexts change. The analysis followed in these existing studies also lacked in further exploration of how individual building feature correlates to energy usage, which can be the key to determining the most cost-effective retrofit measure.

This paper attempts to address these gaps by applying Automated machine learning (AutoML) to estimate the year of construction and energy consumption of residential buildings. Publicly available data was used to extract multi-modality features representing buildings’ spatial, morphological and thermal characteristics. The effects of building features towards energy consumption were further examined using a series of permutation feature importance (PFI) and partial dependence plots (PDP). The results provide a hint on what the most essential features are for energy consumption estimation when data is limited, what are the essential housing characteristics that should be considered for selecting target homes for retrofitting and what changes in material or insulation condition may be altered to improve home energy performance.

### Main contributions of the work

This paper investigated the ranking of housing features in correlation to the building age and energy consumption prediction, based on a systematic approach utilising open-sourced data and autoML. This work aims to answer two main questions: 1) *What type of properties may need retrofit?* 2) *What building elements or features may be prioritised to be retrofitted to reduce energy consumption?* These are answered by:

- Identifying the most important features for building age and energy consumption estimation;
- Investigating the marginal effects of most important features on building age and energy consumption to guide retrofit measure selection.

The paper is structured as follows. Section 2 provides a detailed description of what data has been utilised and what pre-process has taken place in this work. Due to the nature of open-source data, the limitations of the used data are listed, followed by how these limitations may hinder the overall model performance. Section 3 presents the methodology this study followed, detailing how the data is aggregated and sub-sampled, how an autoML system is implemented, and how robustness is tested using a comparative study. A case study was conducted based on residential properties in Sheffield with results and discussion offered in the following section 4.

## Data

This paper mainly used data from two sources: Ordnance Survey (OS) and EPC. The map data is used to describe the spatial and morphological characteristics of the houses, while the EPC provides information relating to housings’ material and insulation conditions. The following sections will explain the procedures of the data collection and pre-processing conducted before model development.

### Spatial and morphological data

The spatial and morphological data this paper used is the OS MasterMap Building Height Attribute products^21^. Table  1 has listed all the features extracted and used to describe the buildings’ morphology.

**Table 1:** List of features based on OS MasterMap, with brief descriptions of what they represent of and how they are calculated.

No.	Variables	Description
1	Total floor area	Area of the building footprint (*a*)
2	Perimeter	Total length of building polygon outline (*p*)
3	Relh2	Relative height from ground to the base of the roof
4	Relhmax	Relative height from ground to the highest part of the building
5	NPI	Normalised Perimeter Index (NPI) calculated by $\frac{2\sqrt{a\pi }}{p}$
6	Vxcount	Number of vertices in building polygon
7	Builtrate	Ratio between all property footprint and postcode area

**Fig. 1:**
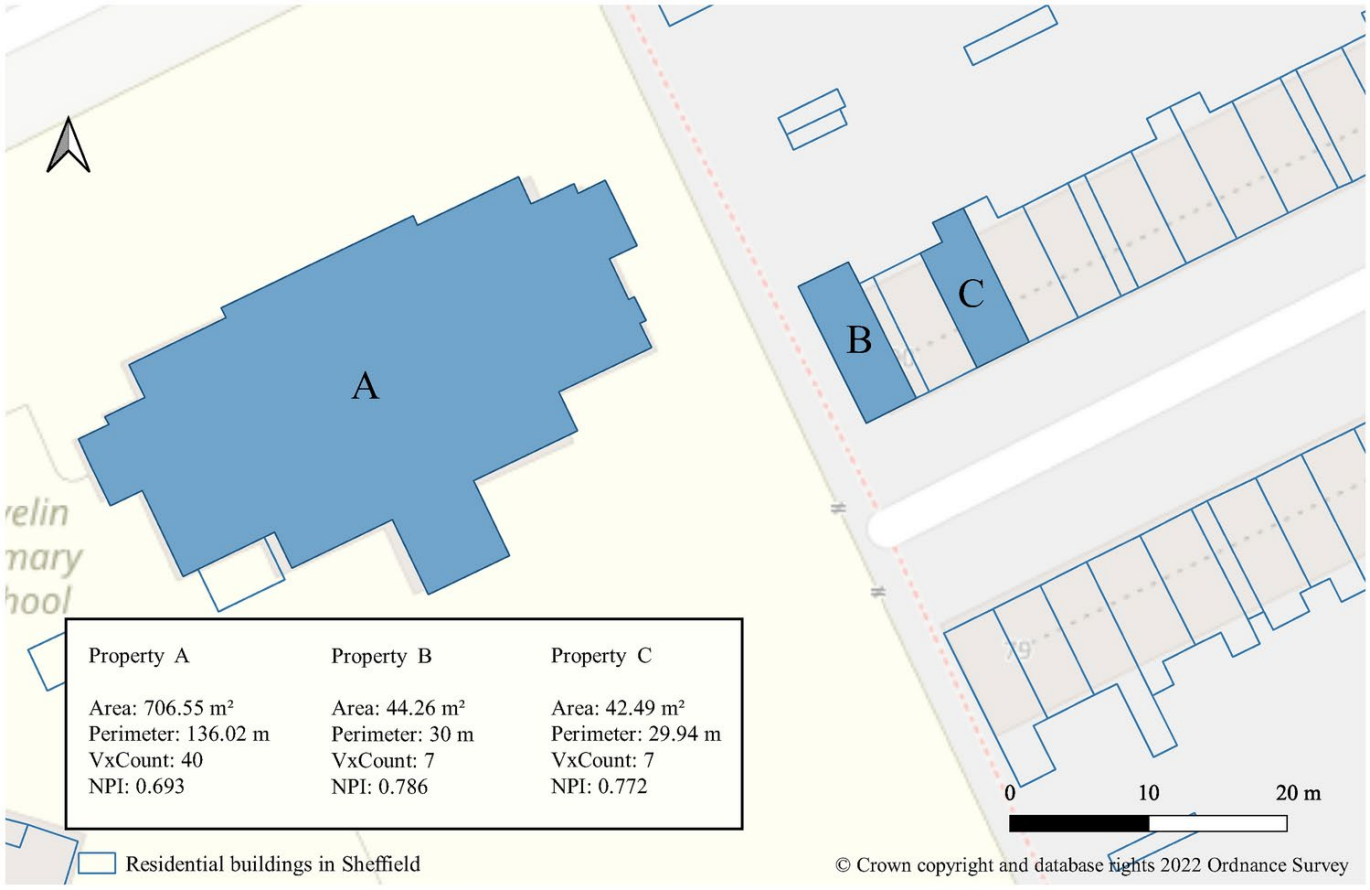
Illustration of example map data.

Variables 1, 3 and 4 are values provided in the OS MasterMap, while the rest are calculated using ArcGIS. Variables 2 and 6 are calculated using the field calculator in Arcmap. Variables 5 and 6 are metrics adapted to describe the complexity of the building shape. Normalised Perimeter Index (NPI) is a shape metric measuring the roundness. An NPI value further departed from 1 suggests the building has a more complex shape^22^. Three properties are highlighted in Figure 1 as an example. Property A is a primary school in Sheffield, while B and C are terraced houses that can be commonly found in the UK. Each property has been marked with its area, total perimeter length and the calculated NPI. By comparing these values, it can be seen that, buildings with more irregular shapes have smaller NPI values. On the other hand, B and C are the same type of houses, so similar values are found for NPI and building perimeter because they are more similar in building shapes.

### Energy performance certificates

In this study, the EPC is used to provide variables relating to buildings’ energy performance. The UK government provides an online database for users to access and download EPC records as spreadsheets^23^. However, as discussed in Section 1.2, studies show that multiple EPC records can be found associated with the same property^18^. This study examined the downloaded EPC, if the property address or reference number occurred multiple times, it means that the property is associated with multiple EPC records. These redundant EPCs are filtered based on when the record was created. The single latest-issued EPC is used as the data input.

Overall, the EPC contains 92 categories offering building-related information from three perspectives: spatial and reference information to identify where the property is (e.g. Unique Property Reference Number (UPRN) and address); the current property characteristics and energy performance; and potential characteristics and energy performance if recommended retrofit implemented. Therefore, a data selection process is essential to filter unnecessary information and avoid high costs in time and computational power. The selected variables and their brief descriptions are listed in Table  2.

**Table 2:** List of data extracted from the EPC, with brief descriptions and example classes in categorical data.

No.	Variables	Description
8	Property type	Type of property (e.g. house)
9	Built form	Type of built-form (e.g. detached)
10	Transaction type	Status in the housing market (e.g. marketed sale)
11	Number habitable rooms	Number of rooms in the property
12	Number heated rooms	Number of rooms that are able to be heated in the property
13	Roof description	Type of roof and its insulation conditions (e.g. pitched)
14	Walls description	Type of walls and its insulation conditions (e.g. filled cavity)
15	Floor description	Type of floor and insulation conditions (e.g. solid, insulated)
16	Lighting description	Percentage of low energy lighting used
17	Main fuel	Type of main fuel used for central heating (e.g. mains gas)
18	Ageband	Construction age grouped in 12 bands (e.g. before 1900)
19	Energy consumption	All energy consumed using fuel and electricity (kWh per year)

Variables 8 to 12 are features describing the general characteristics of the buildings, while variables 13 to 17 provide more detailed descriptions of the conditions of specific building elements. Variable 18 is the completed dataset of ageband combining EPC recorded and predicted year of construction. The original energy consumption recorded in the EPCs is measured in $kWh/m^2$ per year. The total floor area for each house is taken into consideration here to produce variable 19, which is used as the ground truth data for training the energy prediction model. This is to allow future validation with other sources of data, such as smart meter readings and national statistics.

As discussed in the literature review, EPC data can present certain issues. These issues may be caused by the fact that the records were created by multiple inspectors and the use of different versions of EPC guidelines over time, particularly inconsistencies and abnormal entries are found for the categorical variables used in this paper. To address these issues, a two-step processing approach was implemented. The first step is to replace blank or abnormal entries. For example, if the entry is marked as ‘INVALID!‘ or ‘NO DATA‘, these entries are combined as ‘unknown‘. This process also ensures the records only contain English records.

The second step is reorganising the categorical data (variables 13-19). Similar descriptions in the categories are found and merged. For instance, “some double glazed” and “partial double glazed” used to describe the window insulation conditions are combined into one category.

Once the data from OS and EPC are prepared separately, they are matched using the Unique Property Reference Number (UPRN). The UPRN is a reference system commonly found in the UK geospatial data such as the OS map data. It was recently introduced to EPC in November 2021^24^, which enables this paper to match the map data with its corresponding EPC. The combined dataset is then used for training the machine learning models for age and energy prediction, which will be explained in the methodology section.

## Methodology

**Fig. 2:**
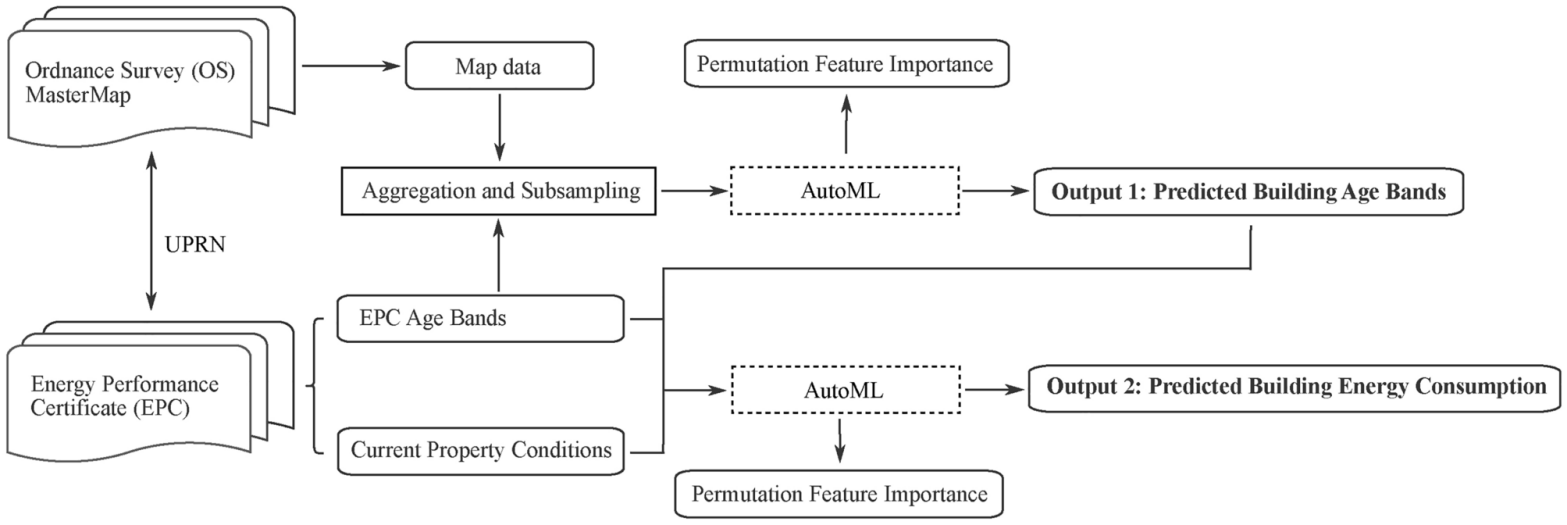
The designed workflow this study follows, including data inputs (OS and EPC), information extraction and pre-processing, model training by autoML and outputs.

This section presents the development of supervised machine learning models for age and energy prediction. The overall workflow is illustrated in Figure 2. The first model trains an autoML to predict construction age bands for properties with no age specified in the EPC. This step ensured the data for energy consumption prediction is complete. The second model then predicts energy consumption based on properties’ morphological and thermal characteristics.

### Age bands aggregation and subsampling

The ground truth data used in training the age prediction model is the age band recorded in the EPC, variable 19 in Table 2. The EPC has 12 age bands in total: before 1900; 1900-1929; 1930-1949; 1950-1966; 1967-1975; 1976-1982; 1983-1990; 1991-1995; 1996-2002; 2003-2006; 2007-2011; 2012 on-wards. These age bands are classified following the changes in regulation for building construction, which mainly are amendments for the conservation of fuels and power^17^. The way the age bands are classified suggests it may not be the best representation of how buildings’ physical shapes and designs change over time. Relatively lower prediction accuracy is expected when conducting the age detection. However, this is the only open-sourced data that can be found offering adequate spatial coverage and level of detail for property age. There are other age data, such as the products from Verisk^25^, which interprets building age from imagery, but classified the age in a very generic way (i.e. historic, postwar and modern).

Although the uneven distribution can be considered as a representation of the number of properties constructed in the real world, it can negatively affect the performance of machine learning models. Machine learning models usually try to maximise the prediction accuracy by assigning more weights to classes with more occurrences^26^. To reduce the bias caused by the imbalanced distribution, age bands with fewer records are aggregated into one class, as explained in section  2.2, and then a simple random sampling method is used to randomly select 4,000 properties from each age band for prediction.

### Automated machine learning

After initially processing the raw input data, the workflow then proceeds to the next stage to train and perform prediction using autoML. The autoML approach can be considered a complete “black box”. It offers a combined algorithm selection and hyper-parameter optimisation tool to reduce the costs of machine learning model development^27^. It takes care of raw data input from the beginning to the final step, offers a tool that reduces development costs, and at the same time ensures optimal estimation accuracy^28,29^. A wide range of open-source autoML tools is available to choose from.^30^ analysed six recent autoML libraries: Auto-Sklearn, AutoGluon, H2O AutoML, rminerAutoML, TPOT and TransmogrifAI. Their performance were tested and compared on binary and multi-class classification and regression-based machine learning tasks using thirteen benchmark datasets. Small differences in prediction accuracy were found among the inspected tools, 3% to 16% difference for binary classification tasks, 4% to 8% for multi-class classification, and only 1% difference was found when training with all regression data^30^. Such little difference suggests that the selection of the autoML tool will have limited impacts on the overall prediction accuracy.

#### Auto-sklearn

**Fig. 3:**
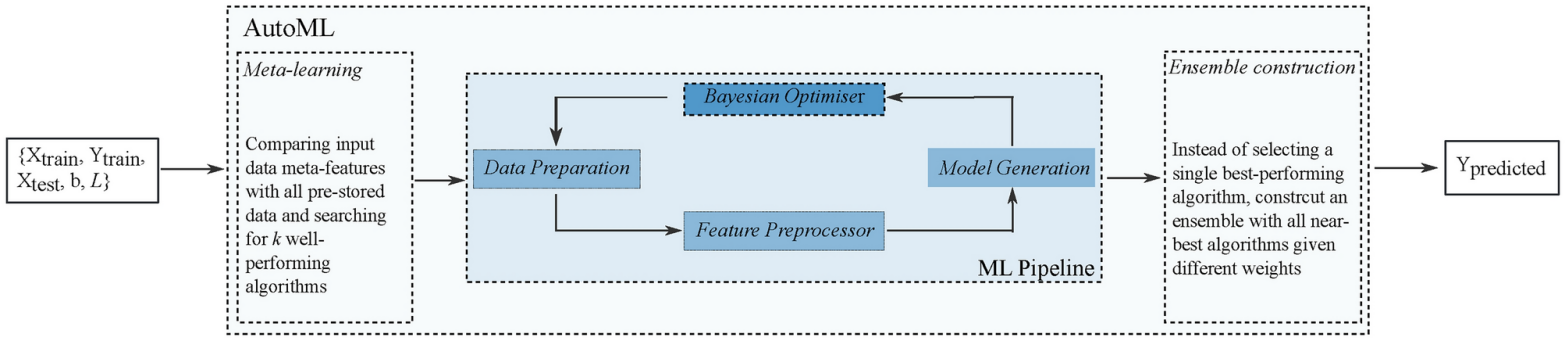
An overview of the Auto-sklearn system. The input data follows the pipeline to construct the most optimal model and then perform prediction. The pipeline involves meta-learning, data preparation, feature preprocessor, model generation, Bayesian optimisation and ensemble construction.

Auto-sklearn was selected as the automated model development tool for this study. Auto-sklearn is an autoML tool developed based on Scikit-learn, a popular Python library offering a wide range of machine learning algorithms^27^. As illustrated in Figure 3, Auto-sklearn can be considered as a pipeline with three main steps. The first step is meta-learning, where the input data is compared with pre-stored benchmark data^27^. Algorithms that performed well on benchmark data that is similar to the user inputs are selected as target algorithms. The second stage then trains, fine-tunes and evaluates all target algorithms. The Bayesian optimisation simultaneously calculates the correlations between the hyper-parameter settings and the prediction accuracy. This correlation is the main criterion the Auto-sklearn used for algorithm selection. The pipeline also tests whether building an ensemble of multiple algorithms will achieve better prediction performance.

Two models were separately trained using Auto-sklearn, a classification model for age band prediction, and a regression model for energy consumption prediction. To minimise the effects of multi-collinearity, the input data were divided into two sets based on the rules stated in Section 1.2. Building age bands were predicted primarily based on the spatial and morphological features of buildings, and energy consumption was predicted with more thermal-related features. When training, all the input data was randomly split, 80% is used for training and 20% for testing. The trained model performance on the new dataset was examined using the testing data.

The performance of all the trained algorithms was evaluated. Model accuracy score and F1-Macro score were used for the age classification model. The accuracy score calculates the proportion of predicted labels that exactly matched with the “true” labels^31^. The most optimal algorithm for age band prediction was then used to predict the construction year band and complete the information for houses without age bands recorded. Regression models for energy consumption prediction were evaluated by $R^2$ and the mean absolute percentage error.

#### Comparison study between Auto-sklearn and traditional ML pipeline

This work also conducted a comparison study as a robustness test to examine whether Auto-sklearn outperforms a traditional machine learning pipeline, one algorithm selection and fine-tuning are conducted in separate steps. Similar to how Auto-sklearn behaves, the input data was preprocessed. Numeric data, variables 1-7, 11, 12, 16 and 19 (in the energy prediction model), was normalised to be unit invariant. Categorical data, variables 8-9, 13-15, 17 and 18, was processed using the one-hot encoding. This encoding process converts each class in the categorical data into separate features in a binary format. If the sample falls into this feature, then 1 is marked, otherwise 0.

A list of algorithms that have been used by existing studies was selected: linear regression^9,20^, K-nearest neighbours^20^, random forest^8,9,13,20^, decision tree^20^ and gradient boosting^20^, were tested for both age and energy consumption predictions. The same evaluation metrics, F1-Macro and $R^2$ score, were applied for evaluation and comparing the performance of models trained using auto-Sklearn.

As shown in Table 3, the traditional pipeline provided a result different from what auto-Sklearn concluded. Among the five algorithms, random forest estimators achieved the best performance for both prediction tasks. It is also the algorithm that most of the existing studies have applied for residential building energy estimation^8,9,13,20^. The resulted predictions are also less accurate than the Auto-sklearn computes.

**Table 3:** Comparison among model training scores for all predictions to check the robustness of using autoML. Different algorithm and better training accuracy were concluded by applying autoML.

		Age bands classification		Energy consumption regression	
Algorithm		Model Score	F1-Macro	$R^2$	MAPE
AutoML	**Gradient Boosting**	**0.543**	**0.540**	**0.828**	**18.1%**
Manual	Linear Regression			0.753	23.9%
	K-Nearest Neighbours	0.412	0.583	0.758	19.1%
	Decision Tree	0.445	0.901	0.554	22.5%
	**Random Forest**	**0.468**	**0.991**	**0.776**	**18.7%**
	Gradient Boosting	0.446	0.473	0.767	20.9%

### Permutation feature importance

Permutation feature importance (PFI) was used to rank how each variable can affect the overall model performance. The PFI is calculated by randomly shuffling or permutating each input data. The resulting prediction accuracy before and after the shuffling are calculated and compared. The larger difference in accuracy score suggests the variable is relatively more important to the model^32^. Compared with the gini feature importance used in the existing study^8^, the PFI performs better in dealing with categorical variables, especially if they are processed with the one-hot encoder. For example, after one-hot encoding procedure, the feature class ‘Property type‘, will be expended into four separate variables: property type: bungalow, property type: flat, property type: house, and property type: maisonette. The gini feature importance can only provides individual measures on the four sub-classes; while the PFI is able to store and permute before they are processed with the one-hot encoding system. More useful hints on what input data in their original class are necessary for the predictions can be offered.

### Partial dependence

To further investigate how the building features contribute to the prediction of each age band and overall energy consumption, partial dependency (PD) are adopted. The PD calculates the average marginal effects a target feature has towards the prediction outcomes^32–34^. For a machine learning model $F\left( \ldots \right)$ trained with features $x_{i}$, each x produces an estimation result $y_{k}$, where $i=1,2,3\ldots ,p$ and $k=1,2,3,\ldots ,N$. The output of this machine learning model can be written as ${\hat{y}}_{k} = F\left( x_{1,k},x_{2,k},\ldots ,x_{p,k} \right)$. The PD $\Phi (x)$ of target numerical variable $x_{j}$ can be calculated using the following equations, where ${\bar{x}}_{i}$ represents the average value of $i^{th}$ covariate^33^:

$$\begin{aligned} \Phi j(x)&=\frac{1}{N}\sum _{k=1}^{N}F(x_{1,k},\ldots ,x,\ldots ,x_{p,k})\\ \Phi j(x)&=a_{j}x + \frac{1}{N}\sum _{k=1}^{N}\sum _{i\ne j} a_{i}x_{i,k}\\&=a_{j}x + \sum _{i\ne j} a_{i}{\bar{x}}_{i} \end{aligned}$$

For categorical variables, the PD replaces all the input features with the target feature and then calculates the average results^32,34^. This value suggests when all other elements remain similar, how the average energy consumption prediction would change relatively when the variable changes to the target feature.

## Case study: residential houses in sheffield

### Overview

This paper has conducted a case study focusing on all residential buildings in Sheffield, UK. Following the steps explained in the data and methodology sections, EPC records for all residential buildings in Sheffield available as of December 2021 were downloaded. All these records were first filtered so every property only contains the latest record. Among all EPCs downloaded, there were 23.5% properties found to be associated with multiple records which add up to 34.3% EPC records. The resulting dataset comprised 142,756 homes and their associated EPC records for the following study. According to the EPC, the residential properties in Sheffield have an average energy consumption of around 274.50 kWh/$\hbox {m}^2$ per year or 22219.42 kWh per year, if the footprint for each property recorded in the EPC is used for calculation.

**Fig. 4:**
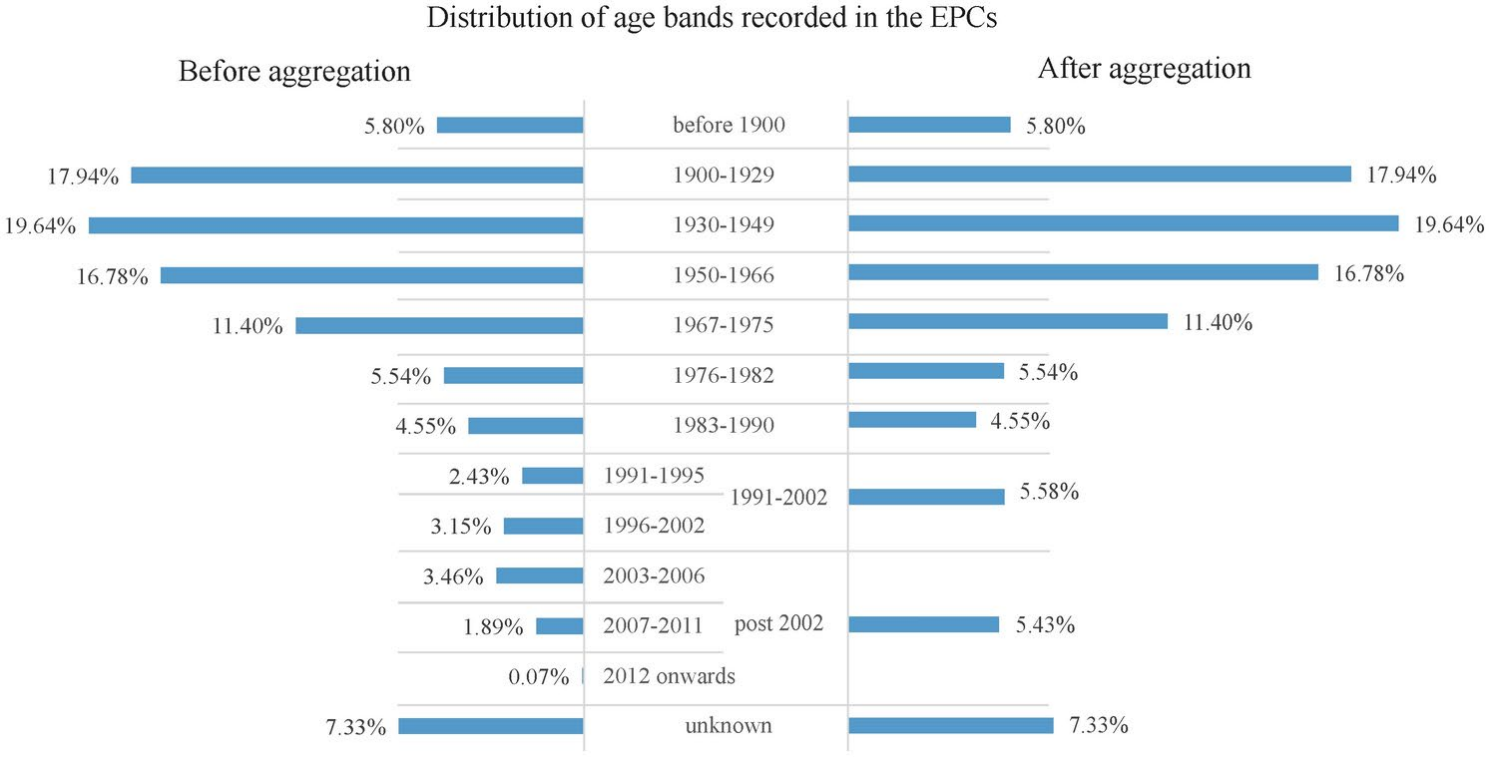
Distribution of construction age recorded in the EPCs before (left) and after aggregation (right).

As illustrated in Figure  4, before aggregation, the original records from EPCs show that most of the residential buildings in Sheffield were developed between 1900 and 1966, and few were built after 2012. There are also 10,392 (7.3%) properties whose construction age remains unknown. Without pre-processing, this uneven distribution will lead to a biased model. Based on the number of properties each age band contains, and how EHS classifies the age band groups^5^, the age band 1991-1995 and 1996-2002 were combined into the new class “1991-2002”; 2002-2006, 2007-2011 and 2012 on-wards were aggregated into the new class “post-2002”. The aggregation process ensured all age bands had enough data to follow the sampling process for model training.

Table 4 summarises the basic statistics of the numeric data and their subsets used in predictions, including their average, standard deviation (std) and coefficient of variance (CV). The summary of categorical data used in this paper is included in the Appendix in the supplementary material. The last four variables in Table 4 are only used for energy prediction so no subsamples were generated. The coefficient of variance is calculated as the ratio between the std and the mean. Among all the numerical data used in this study, it is not surprising to find that, except for the built rate, all the variables have CV less than 1. As more than 70% of residential properties in Sheffield are houses, they tend to have relatively similar physical features, the same as the example map illustrated in Figure 1. The only variable that has a CV larger than 1 is the built rate indicating a high variability in building distribution across Sheffield. For instance, properties in rural areas near the Peak District are less densely built compared to those in neighbourhoods closer to the city centre. By comparison, the subsets generated using the sampling method can to some extent be considered representative of all the data collected, as there is no significant difference between the statistics of original and subsampled data. In comparison, the subsets generated using the sampling method can be considered reasonably representative of the entire dataset, as no significant differences were observed between the statistics of the original data and the subsampled data.

**Table 4:** Statistics of numeric data used for model prediction, before and after applying the simple random sampling approach.

Variables	All Samples			Subsamples		
	Mean	Std	CV	Mean	Std	CV
Total floor area	81.45	38.16	0.47	81.02	40.11	0.49
Perimeter	41.82	26.01	0.62	45.84	32.83	0.72
Relh2	6.33	3.26	0.52	6.78	3.95	0.58
Relhmax	8.17	3.40	0.42	8.73	4.16	0.48
NPI	0.78	0.04	0.05	0.77	0.05	0.06
Vxcount	12.57	7.29	0.58	9.96	5.00	0.50
Builtrate	0.21	0.28	1.33	0.23	0.36	1.57
Number habitable rooms	4.06	1.77	0.44			
Number heated rooms	3.96	1.76	0.44			
Lighting description	0.53	0.34	0.64			
Energy consumption (kWh)	22219.42	14149.90	0.64			

### Results of model training and prediction

#### Age detection

The age detection model was trained on the processed dataset. The auto-Sklearn detected 37 algorithms that might be optimal for predicting building age bands. The most optimal model used a gradient boosting algorithm, which trains the model by sequentially adding input variables to the ensemble of decision trees and refitting the model based on the errors made by the previously added inputs^35^.

**Fig. 5:**
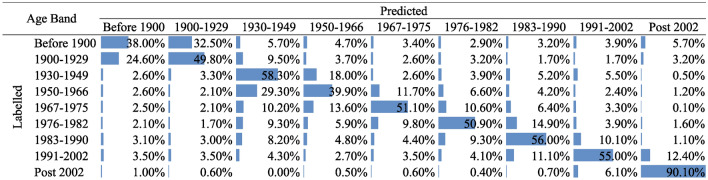
Heatmap table showing the resulted between the true (columns) and predicted age bands (rows) using the random forest classification.

For the testing data, the most optimal model Auto-Sklearn trained achieved an accuracy score of 0.543 and an F1-Macro score of 0.540. The model performance was further evaluated by comparing the predicted age bands with their true class in EPC records. As illustrated in Figure  5, the accuracy score suggests that the majority of the properties are correctly predicted, especially for the aggregated age bands, for post-2002, 90.10% properties were predicted correctly. However, mis-classifications were observed, for instance, only 38% of properties built before 1900 were correctly predicted.

One potential reason, as discussed in the data section, is that the age bands are classified based on the changes in energy regulations, the errors are to some extent expected. Another possible reason could be that property developers tend to design houses that fit into the general architectural styles of neighbouring properties, which may not reflect the actual construction period. Additionally, inaccuracies might arise from incorrect labelling by the EPC inspectors.

#### Energy consumption prediction

The energy consumption prediction was then conducted after age bands were classified for each housing. The age prediction results from the first model were used to train the model. Auto-sklearn determined the best-performing algorithm used data preprocessors based on feature type, feature agglomeration as feature processors and gradient boosting as the regressor. The trained model achieved a $R^2$ score of 0.828, and a mean absolute percentage error (MAPE) of 18.1%. The results suggest that overall, around 82.8% of the test data can be explained by the trained algorithm; and the prediction results based on the test data have an average difference of 18.1% compared with the ground truth.

### Feature importance

**Fig. 6:**
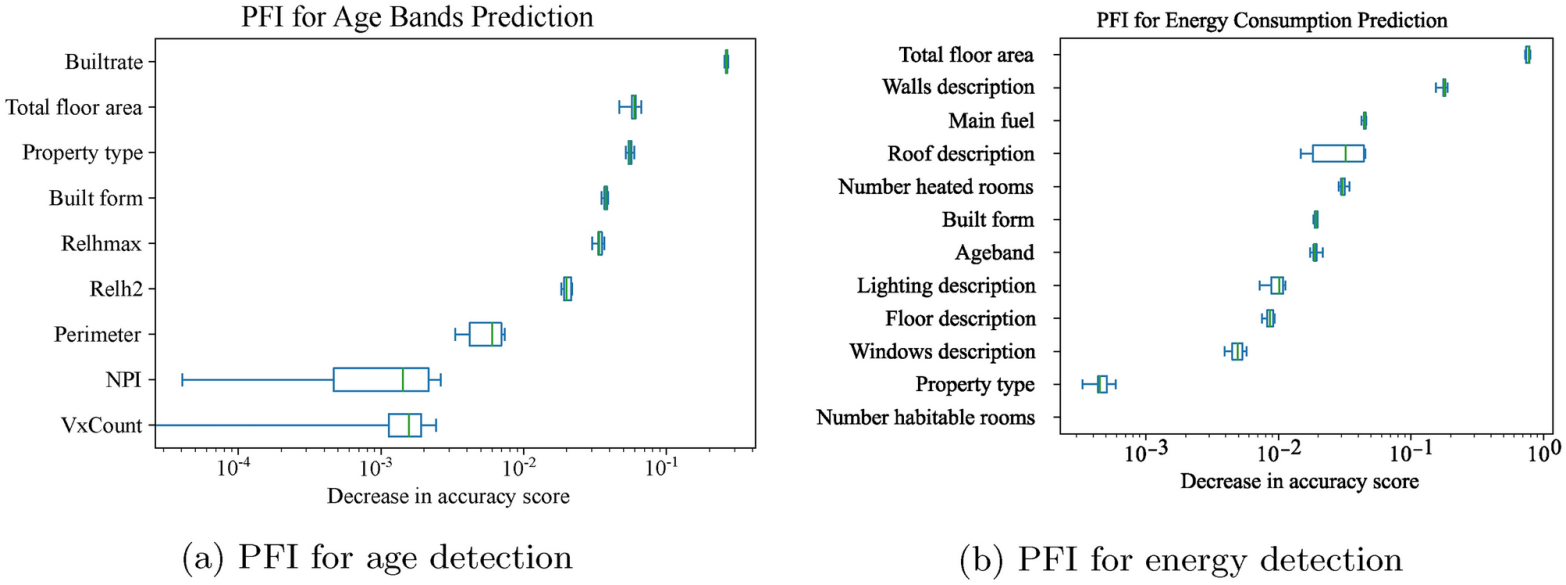
PFI for variables used in the two machine learning models, x-axis in log form. (**a**) is for age detection and (**b**) is for energy consumption prediction.

The PFI plotted in Figure 6 ranked how important each input feature is in both models towards the prediction. The x-axis is plotted in its log form, to offer clearer visualisation for variables with less feature importance.

The features used for the age prediction model are ranked in Figure 6a. The importance rank suggested that, the built-up rate is the most important feature when predicting the age bands of residential buildings in Sheffield, floor area and property types are also relatively important. Excluding the variable builtrate caused a 23.9% decrease in model accuracy score, and a 25.6% decrease in F1-Macro score.

The NPI and the number of vertices are found relatively less important. As the example properties illustrated in Figure 1, when predicting the age of residential buildings, buildings tend to have little difference in shapes and thereby less sparsity in values can be found. Excluding NPI and the number of vertices only caused a decrease in accuracy score and F1-Macro by 0.37% and 0.56% respectively. Overall, when data availability is limited, the age band of the housing can be estimated by gathering information on the housing size, the building type, and how densely the postcode area is developed.

Figure 6b ranked how the input data affect the model performance when estimating energy consumption for Sheffield. The total floor area is the dominating feature in this estimation, followed by building materials, which is also the most common retrofit target. Excluding total floor area from model training led to a 15.3% decrease in $R^2$ score and a 26.0% increase in MAPE value.

On the other hand, the type of property and number of habitable rooms are less important in estimating housing energy consumption, excluding these features only resulted in 2.80% decrease in $R^2$ score and 3.26% increase in MAPE. Houses’ age bands ranked seventh among all features, which indicates that it has relatively less impact on energy consumption prediction.

### Partial dependence

#### Age detection

**Fig. 7:**
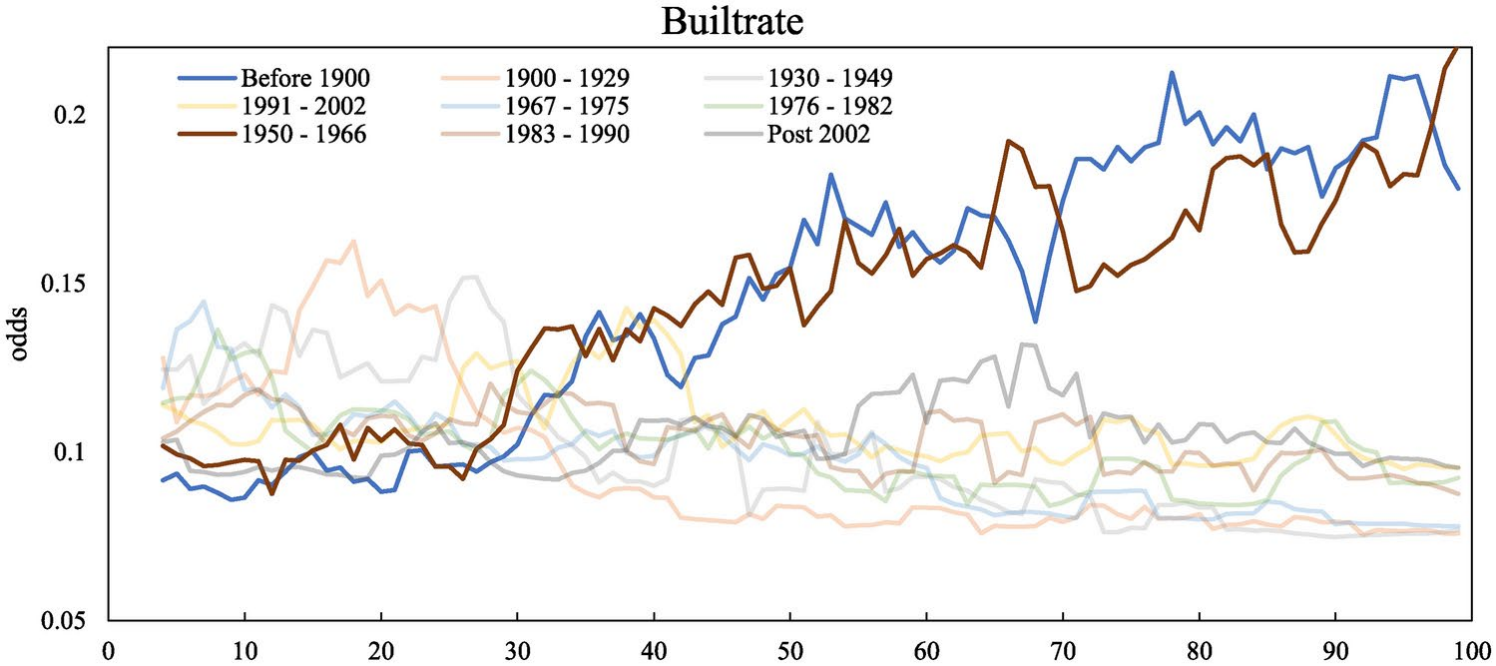
Partial dependence plot of built rate (x-axis) against the possibility of being built in the target ageband (y-axis).

According to the feature importance calculated in the last section, the built rate is the key feature when estimating housing age. Figure 7 illustrates the complex relationships between the built rate and each age band in Sheffield. The trendlines showing positive correlations are highlighted while negative correlations are light-coloured. In general, postcode areas with a built rate of less than 30% tend to have a combination of houses built in different eras. If the area has a built rate higher than 30%, houses in the area are more likely built before 1900 or between 1950 and 1966.

#### Energy consumption prediction

**Fig. 8:**
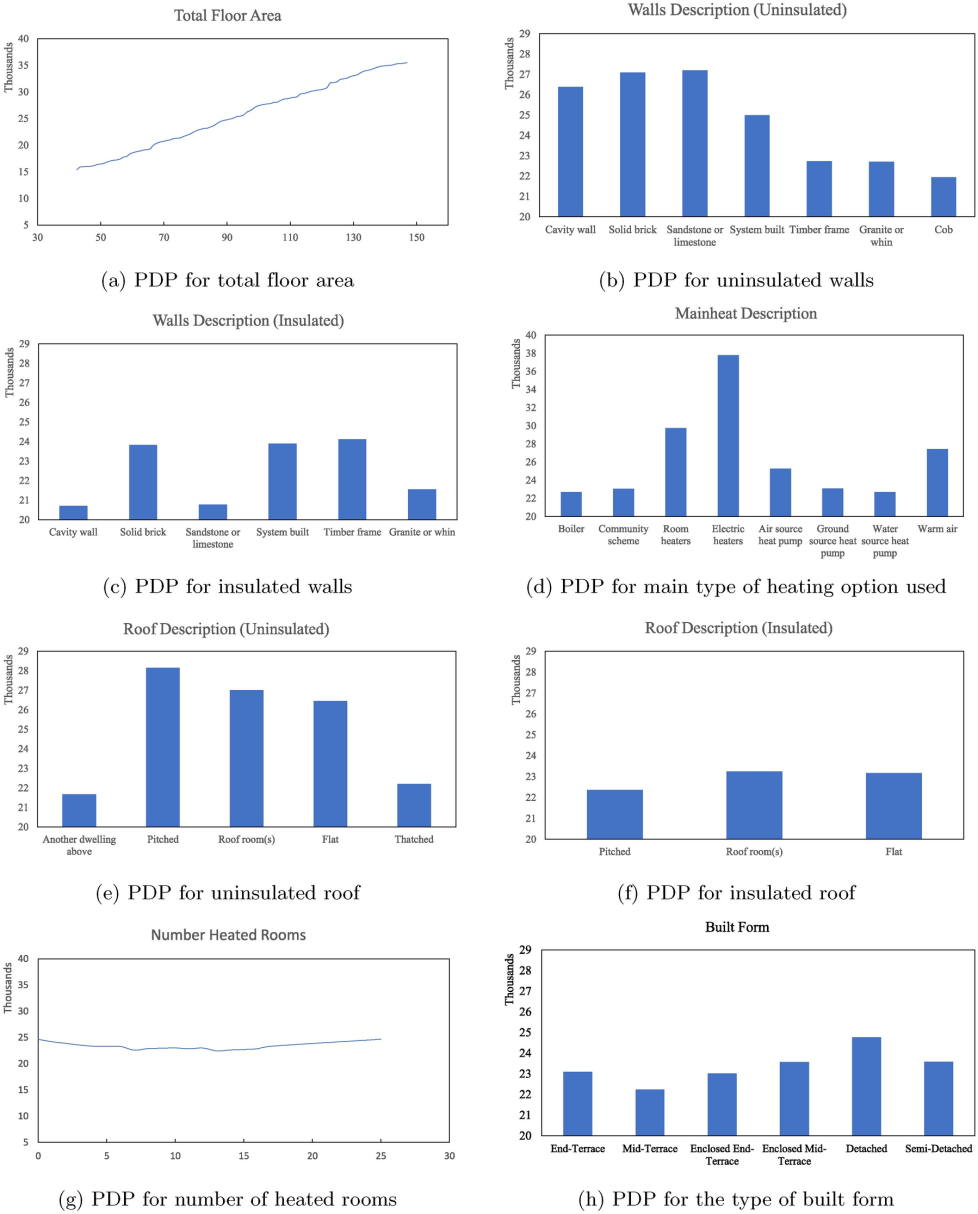

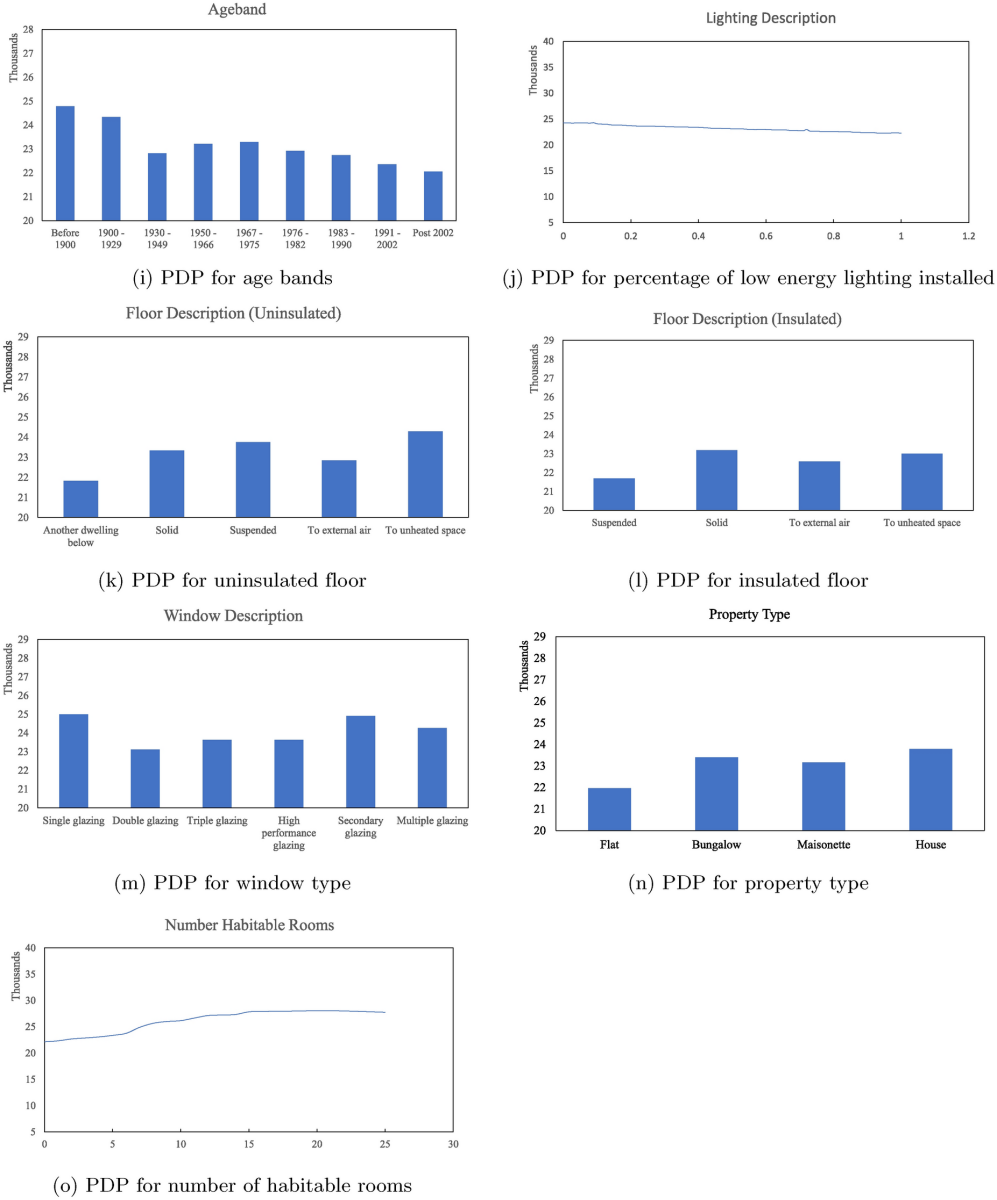
PDP for the marginal effects of building features (the x-axis) towards residential energy consumption in kWh (the y-axis) in Sheffield.

The partial dependence plots in Figure 8 show how each building feature affects energy consumption. The charts are ordered according to their ranked permutation feature importance. For features referring to the building fabric: walls, floors, and roofs, separate charts are produced to indicate whether insulation is installed and the corresponding energy consumption variations.

The dominant feature ranked by PFI in Figure 6b is the total floor area of the house. A positive linear relationship can be found between house sizes and energy consumption, as shown in Chart 8a. In other words, in general, larger houses in Sheffield usually have higher energy consumption. A similar correlation can be found for the number of habitable rooms in chart 8o, as it is also an indicator of how large the property is, its importance is weakened in the overall energy prediction and ranked lowest in the calculated PFI. On the other hand, the number of heated rooms in chart 8g has less impact on the model prediction accuracy. The energy consumption firstly slightly decreases when more rooms can be heated in the property, then stays relatively stable. As this number only refers to the number of rooms with heating facilities, it does not necessarily suggest the number of heatings in use, thereby the energy consumption does not change significantly.

The building fabric is the next key feature in estimating housing energy consumption. How different types and conditions of housing material may affect housing energy consumption are intensively researched^36,37^. Charts 8b, 8c, 8e, 8f, 8j, 8k, 8l, and 8m provided a comparison to show how different material used for each building element may affect the energy consumption in Sheffield.

Closer inspection of Figure  8b and Figure  8c shows that, in general, insulated walls perform significantly better in energy saving than uninsulated ones. Without insulation, houses with walls built in timber frame, granite or whin or cob may have less energy consumption. Although all uninsulated walls should be upgraded to reduce heat loss, houses built with cavity walls and sandstone or limestone walls may witness a relatively significant reduction in energy consumption.

The overall energy consumption can also be reduced significantly by applying insulations to the roof and floor. Among the five types of roofs in Figure 8e, without insulation, pitched roofs tend to have higher energy consumption. After insulation, different roof types are likely to perform similarly. For the floor, floors connected to unheated space or suspended should be prioritised for retrofitting.

The energy consumption of residential buildings in Sheffield also has a positive relationship with the proportion of low-energy lights and energy-effective windows. In general, more low-energy lights installed, and better window material (e.g. double and more glazing) used means less energy consumption.

How different heat sources may affect energy consumption are compared in Figure  8d. According to the chart, houses that use electric heaters tend to have the highest energy consumption in Sheffield, while houses with different kinds of heat pumps use less energy. One interesting finding is that boilers were found consume less energy than air source heat pump and electric heaters. This finding is to some extent contradictory to existing studies, where boilers are usually found to be less energy efficient^38^. This observation may be a result of how the heating systems are recorded in the EPCs. Most certificates only contain records of the primary heating source, as the category name “MAINHEAT DESCRIPTION” suggests^23^. For instance, properties with multiple heating options may only list one of them as the primary source, because they are more frequently used or more prominently installed in specific areas of the property (e.g., bedrooms or smaller spaces). It is important to note that this prediction does not evaluate the efficiency of heating sources but rather the total aggregated energy usage. Therefore, this ranking may be suggesting that people living in boiler-installed accommodations in Sheffield consume less energy comparing to others. This interpretation could be misleading for retrofit decisions, and will be addressed further in the discussion section.

Charts for the remaining housing features: the built form in Figure 8h, the age band in Figure 8i and the property type in Figure 8n, may provide preliminary guidance on the potential housing that should be targeted for home upgrade projects. Together with the relationship shown in Figure 8a, the model suggests that larger and older detached houses in Sheffield consume more energy, and may be prioritised for retrofit.

## Discussion

This paper presented a robust model development approach with AutoML application. The comparison with traditional machine learning pipelines and the resulting $R^2$ score of 0.828 further emphasised the efficiency and reliability of AutoML. This application suggests that AutoML could serve as a practical tool for practitioners aiming to develop rapid and scalable energy prediction models.

The findings from the two models have important implications for rapid assessment for retrofit strategies. The key features influencing model performance identified in Figure 6, i.e. Built rate for property age description, Total floor area and Walls description for energy consumption prediction, emerge as critical predictors and should be prioritised in data curation to enhance the robustness and reliability of predictions in related research and applications.

The partial dependence plots in Figure 8 explored the relationship between building materials, insulation, and energy consumption for residential properties in Sheffield. Significant reductions in energy consumption can be seen when insulations are present. Comparisons between different materials and heating sources also inform the optimal choices to reduce energy consumption. In this study for Sheffield, the analysis revealed that larger houses and older detached properties are more likely to consume more energy (Figure 8i,  8a, and  8n), and also highlights the potential of targeted retrofit programs focusing on external walls and roofs to yield maximum efficiency gains (comparison from Figure 8c and  8b, Figure 8f and  8e). This finding corresponds with the recent statistics provided in the National Energy Efficiency Data-Framework (NEED)^39^, where at the national level, NEED found that “Median domestic electricity and gas consumption is higher for larger properties and increases with adult occupancy.”, and insulations for solid walls and cavity walls would provide the highest and consistent savings in gas consumption.

### Limitation and recommendations

Despite this paper has strengths in providing timely research for all the residential buildings in Sheffield, the reliance on Energy Performance Certificate (EPC) data introduces potential inaccuracies in multiple perspectives. The primary limitation is the inconsistencies in the process of producing such certificates. The fact that the records are subjective to the EPC inspectors can significantly affect the interpretability of results. For example, in this paper, the record in “MAINHEAT SOURCE” leads to a contradictory finding with existing research. Future studies may consider using additional sources of data as validation for the records in EPC, such as images and 3D models of the building. The current trends of developing digital twins also offer opportunities for more comprehensive databases to be built for more reliable studies.

Additionally, the study mainly incorporated publicly available data, future studies could consider including behavioural factors, such as occupant energy usage patterns, as it can significantly impact consumption. The finding that properties with boilers consume less energy than other more energy efficient heating sources in this paper may be a result of different occupant behaviour. This limitation restricts the ability of the model to provide a holistic understanding of energy demand. One approximate data that can be used to reflect different occupant behaviour is the smart meter data. While access to smart meter data is strict, future work could acknowledge the impacts and explore the importance of occupant energy usage behaviour by assessing the difference between official statistics, such as EHS and NEED.

Since the primary focus of this paper is to capture the aggregate energy consumption of residential properties, energy usage in kWh was chosen as the prediction output, future research could incorporate energy intensity metrics (in $kWh/m^2$) to normalize energy usage by property size. This would be particularly valuable when assessing the efficiency of heating sources, addressing potential biases and providing more accurate insights into the relationship between heating systems and overall energy performance, supporting more targeted and effective retrofit decisions.

## Conclusion

This paper examined how spatial, morphological and thermal characteristics of residential houses contribute to housing age and energy consumption prediction, by applying an automated approach in machine learning model development. The trained model achieved a $R^2$ score of 0.828 in predicting residential building energy prediction. The permutation feature importance plots offered hints in the essential information required for each model when data availability is limited to perform prediction. That means, when SAP calculation is not available, this approach can be followed to obtain a relatively accurate understanding of the building energy consumption using variables with higher rank of feature importance: housing size, material and conditions of the external walls, and also the main fuel used.

The inclusion of permutation feature importance and partial dependence analyses provided an explainable framework for understanding feature contributions. The analysis highlights the importance of addressing larger, older detached properties with insulation upgrades for walls, as the model results suggest these would yield the most significant reductions in energy consumption.

The limitations of this work were acknowledged, especially the reliance on EPC data, which introduced biases to the model. Future research can be conducted to explore potential additional or alternative data sources to describe the building’s thermal and physical conditions. For example, photos of the target properties and 3D building models. Including data describing energy intensity and energy user behaviour in future research would also further enhance the robustness of energy prediction and support the development of more comprehensive retrofit strategies. This study lays the groundwork for leveraging machine learning in energy efficiency studies and highlights pathways for energy consumption reduction.

## Supplementary Information

Supplementary Information.

## Data Availability

The datasets generated during and/or analysed during the current study are available from the corresponding author on reasonable request. Basic statistics are included in the manuscript as appendix in the supplementary material.
