# Supplementary material for: City-scale residential energy consumption prediction with a multimodal approach
Source: Sci Rep. 2025 Feb 13;15:5313. doi: 10.1038/s41598-025-88603-2 (PMC11821810; doi:10.1038/s41598-025-88603-2)
Supplement: Supplementary file 1 — Supplementary Information. [file 41598_2025_88603_MOESM1_ESM.pdf]

Supplementary Material: City-scale residential energy consumption prediction with a multimodal approach

Yulan Sheng[a*], Hadi Arbabi[b], Wil OC Ward[c], Mauricio A Álvarez[d] and Martin Mayfield[b]

[a] School of Earth and Environment, University of Leeds, UK

[b] School of Mechanical, Aerospace and Civil Engineering, The University of Sheffield, UK

[c] School of Computing and Mathematical Sciences, University of Leicester, UK

[d] Department of Computer Science, University of Manchester, Manchester, UK

[*] Corresponding email: y.sheng1@leeds.ac.uk

Appendix: Statistics of categorical data used in case study

Total sample size: **142,756** residential houses

Table 1: Property type

Property type	Count	Proportion
Bungalow	6,481	4.54%
Flat	31,428	22.09%
House	101,220	70.84%
Maisonette	3,627	2.54%

Table 2: Built form

Built form	Count	Proportion
Detached	24,808	17.40%
Enclosed End-Terrace	1,654	1.17%
Enclosed Mid-Terrace	1,177	0.83%
End-Terrace	20,323	14.22%
Mid-Terrace	41,763	29.21%
Semi-Detached	49,363	34.53%
unknown	3,668	2.66%

Table 3: Floor description

Floor description	Count	Proportion
(another dwelling below)	23,045	16.20%
Conservatory	2	0.00%
Solid, insulated	4,827	3.37%
Solid, uninsulated	26,698	18.67%
Suspended, insulated	4,172	2.92%
Suspended, uninsulated	68,153	47.67%
To external air, insulated	154	0.11%
To external air, uninsulated	163	0.11%
To unheated space, insulated	1,642	1.15%
To unheated space, uninsulated	6,451	4.51%
Average U-Value 0-1.33	7,399	5.23%
unknown	50	0.04%

Table 4: Windows description

Windows description	Count	Proportion
Double glazing	129,050	90.39%
High performance glazing	7,799	5.47%
Multiple glazing	192	0.13%
Secondary glazing	591	0.41%
Single glazing	4,831	3.38%
Triple glazing	195	0.14%
unknown	89	0.08%

Table 5: Walls description

Walls description	Count	Proportion
Cavity wall, insulated	75,517	52.82%
Cavity wall, uninsulated	18,479	12.92%
Cob, as built	10	0.01%
Granite or whin, insulated	17	0.01%
Granite or whin, uninsulated	186	0.13%
Sandstone or limestone, insulated	648	0.45%
Sandstone or limestone, uninsulated	6,833	4.78%
Solid brick, insulated	1,381	0.97%
Solid brick, uninsulated	23,085	16.15%
System built, insulated	2,479	1.73%
System built, uninsulated	1,485	1.04%
Timber frame, insulated	2,010	1.41%
Timber frame, uninsulated	121	0.08%
Average U-Value 0-2.1	10,458	7.46%
unknown	47	0.04%

Table 6: Roof description

Roof description	Count	Proportion
(another dwelling above)	20,833	14.63%
Flat, insulated	2,948	2.06%
Flat, uninsulated	1,971	1.38%
Pitched, insulated	83,452	58.37%
Pitched, uninsulated	21,332	14.92%
Roof room(s), insulated	2,566	1.79%
Roof room(s), uninsulated	2,006	1.40%
Thatched	5	0.00%
Thatched, insulated	9	0.01%
Average U-Value 0-2.4	7,560	5.37%
unknown	74	0.06%

Table 7: Main fuel

Main fuel	Count	Proportion
biogas	9	0.01%
biomass	35	0.02%
coal	150	0.10%
dual fuel	64	0.04%
electricity	10,242	7.19%
from heat network	3	0.00%
gas	130,669	91.43%
LPG	146	0.10%
no heating	409	0.29%
oil	165	0.12%
waste combustion	210	0.15%
wood	16	0.01%
unknown	638	0.54%
